# Hybrid Magnetic Cross-Linked Enzyme Aggregates of Phenylalanine Ammonia Lyase from *Rhodotorula glutinis*

**DOI:** 10.1371/journal.pone.0097221

**Published:** 2014-05-13

**Authors:** Jian dong Cui, Li li Cui, Song ping Zhang, Yu fei Zhang, Zhi guo Su, Guang hui Ma

**Affiliations:** 1 Research Center for Fermentation Engineering of Hebei, College of Bioscience and Bioengineering, Hebei University of Science and Technology, Shijiazhang, P R China; 2 National Key Laboratory of Biochemical Engineering, Institute of Process Engineering, Chinese Academy of Sciences, HaiDian district, Beijing, P R China; 3 Key Laboratory of Industry Microbiology, Ministry of Education, Tianjin University of Science and Technology, Tai Da Development Area, Tianjin, P R China; 4 Collaborative Innovation Center of Chemical Science and Engineering (Tianjin), Tianjin, China; Glasgow University, United Kingdom

## Abstract

Novel hybrid magnetic cross-linked enzyme aggregates of phenylalanine ammonia lyase (HM-PAL-CLEAs) were developed by co-aggregation of enzyme aggregates with magnetite nanoparticles and subsequent crosslinking with glutaraldehyde. The HM-PAL-CLEAs can be easily separated from the reaction mixture by using an external magnetic field. Analysis by scanning electron microscopy (SEM) and confocal laser scanning microscopy (CLSM) indicated that PAL-CLEAs were inlayed in nanoparticle aggregates. The HM-PAL-CLEAs revealed a broader limit in optimal pH compared to free enzyme and PAL-CLEAs. Although there is no big difference in *K_m_* of enzyme in CLEAs and HM-PAL-CLEAs, *V_max_* of HM-PAL-CLEAs is about 1.75 times higher than that of CLEAs. Compared with free enzyme and PAL-CLEAs, the HM-PAL-CLEAs also exhibited the highest thermal stability, denaturant stability and storage stability. The HM-PAL-CLEAs retained 30% initial activity even after 11 cycles of reuse, whereas PAL-CLEAs retained 35% of its initial activity only after 7 cycles. These results indicated that hybrid magnetic CLEAs technology might be used as a feasible and efficient solution for improving properties of immobilized enzyme in industrial application.

## Introduction

In the past twenty years, a carrier-free immobilized enzyme strategy, cross-linked enzyme aggregates (CLEAs), has attracted increasing attentions due to its simplicity in preparation and robustness of the immobilized enzymes. Moreover, CLEAs have a number of interesting features such as highly concentrated volumetric activity, high stability against denaturing agents, low production cost, easiness of synthesis, recoverability/repeated usage, and the fact that no purified enzymes are needed [1]–[3]. So far, CLEAs of various enzymes such as penicillin acylases [4], lipases [5], laccases [6], and alcohol dehydrogenase [7] have been prepared. However, CLEAs technology has also some disadvantages, such as, the particle size of CLEAs is usually small (below 10 µm), which results in difficulties in recovering CLEAs from the reaction medium. Moreover, they may still be considered too soft for many industrial applications [8]–[11]. In order to overcome these problems, some researchers have developed supported CLEAs strategies as alternative solutions to get a biocatalyst with good mechanical properties [12]–[14]. For example, β-glucosidase was immobilized onto mesocellular silica foams (MCF) using enzyme adsorption followed by glutaraldehyde (GA) crosslinking. The resulting CLEAs were proved to be active and recyclable up to 10 cycles without much loss in activity [15]. Carbonic anhydrase (CA) derived from *Rhodobacter sphaeroides* was immobilized on electrospun polystyrene/poly (styrene-co-maleic anhydride) (PS/PSMA) nanofibers as CLEAs. The CA-CLEAs maintained more than 94.7% of its initial activity during 60 days of storage period at 4°C and also retained more than 45.0% activity after 60 times reuses [16]. Although the supported CLEAs strategies have advantages, the volumetric activity of the biocatalyst and productivity of the reaction can be reduced simply due to the presence of the noncatalytic mass of the carrier.

Generally, the use of magnetic materials for the immobilization may improve overall enzyme quality with the following advantages: ease of recovery from a reaction mixture by using an external magnetic field, improving stability for repeated usage in continuous bioseparation processes, and a greater control over the catalytic process [17]–[19]. It was reported that when α-chymotrypsin (CT) was immobilized in magnetic hierarchically-ordered mesocellular mesoporous silica (HMMS) by adsorption of enzymes and subsequent enzyme crosslinking (CLEA-M-CT), the resulting CLEA-M-CT could maintain their initial activity not only under shaking (250 rpm) for 30 days, but also under being recycled for 35 times [20]. Magnetic cross-linked enzyme aggregates of α-amylase were also prepared by chemical cross-linking the enzyme aggregates with amino functionalized magnetite nanoparticles. The magnetic α-amylase CLEAs retained 100% of its initial activity even after 6 cycles of reuse [21]. These results indicated that the strategy of magnetic CLEAs opened an attractive way towards effectively improving catalytic properties of CLEAs.

Phenylalanine ammonia lyase (PAL, EC 4.3.1.24) is an enzyme that catalyzes a reaction converting L-phenylalanine to ammonia and trans-cinnamic acid [22]. However, the production of L-phenylalanine from trans-cinnamic acids was of limited success, partly because of the relatively low specific activity and instability of PAL during the bioconversion [23], [24]. Recently, the preparation of cross-linked enzyme aggregates of crude PAL from *Rhodotorula glutinis* (*R. glutinis*) (PAL-CLEAs) was first introduced by our group. Compared to the free enzyme, the PAL-CLEAs showed increased thermal stability, storage stability and operational stability [25]. However, the particle size of the resulting PAL-CLEAs was still small, which led to difficulty in recovering of the CLEAs particles. In order to overcome this problem, in this work, PAL from *R. glutinis* was co-aggregated with magnetite nanoparticles and subsequently cross-linked with glutaraldehyde to obtain a new hybrid magnetic PAL-CLEAs (HM-PAL-CLEAs). The optimal catalytic temperature and pH as well as the stability of HM-PAL-CLEAs were fully investigated. Lastly, the reusability of HM-PAL-CLEAs was also measured.

## Materials and Methods

### Microorganism and Chemicals

The strain of *R. glutinis* (CICC 32917) was purchased from China Center of Industrial Culture Collection (CICC, Beijing, China). Glutaraldehyde and trans-cinnamic acid were purchased from Sigma (St. Louis, USA). L-phenylalanine was obtained from Beijing Chemical Reagent Company (Beijing, China). Other reagents used were of analytical grade.

### Preparation of Magnetite Nanoparticles

Magnetite particles were prepared according to a previous report [26]. Briefly, 1.25 g of FeCl_2_·4H_2_O and 3.40 g of FeCl_3_·6H_2_O were dissolved in 100 ml de-ionized water at 60°C; then, 6 ml of 25% NH_3_·H_2_O was added and the reactant mixture was vigorously stirred at 180 r/m for 30–40 min. After the color of bulk solution turned to black, the magnetite precipitates were separated and washed several times with the deionized water until a pH value of 7.0 was obtained.

### Production of PAL in *R. glutinis*

The strain of *R. glutinis* (CICC 32917) was cultivated by the method described by Zhang et al [27]. The cells collected by centrifugation were disrupted by glass beads. The cell extracts were centrifuged at 10,000×*g* for 15 min to obtain crude PAL for the preparation of CLEAs and HM-PAL-CLEAs.

### Preparation of PAL-CLEAs and HM-PAL-CLEAs

PAL-CLEAs were prepared according to the procedure reported by Cui et al. [25]. Ammonium sulfate was added up to the final concentration of 55% saturation in crude PAL solution (final proteins concentration of 1.8 mg/ml, about 1.2 U PAL activities) to precipitate the enzyme. After keeping the mixture with stirring for 1 h at 4°C for complete precipitation of enzyme, glutaraldehyde was added to the final concentration of 0.05% (v/v) and stirred for 2 h at 4°C under shaking at 200 rpm. Then the suspension was centrifuged at 10,000*×g* for 10 min at 4°C. The pellet was washed three times in 25 mM Tris-HCl buffer solution (pH 8.8), after which no protein was detected in the washing solution, and finally the prepared PAL-CLEAs was stored in 5 ml Tris-HCL buffer (25 mM, pH 8.8) at 4°C. The amount of cross-linked enzyme was determined by subtracting the amount of enzyme found in washing solutions from the total quantity initially added for immobilization. The protein concentration was measured by Bradford methods using BSA as standard [28].

For HM-PAL-CLEAs preparation, the different amount of the magnetite nanoparticles (mg) was mixed with crude PAL solution (mL) in 25 mM Tris-HCl buffer solution (pH 8.8) and shaken at 4°C. Then the samples were added into 55% ammonium sulfate saturation with shaking. Afterthat, all other procedure was the same as that for the PAL-CLEAs preparation. After cross-linking, HM-PAL-CLEAs were separated using magnet, washed for three times by 25 mM Tris-HCl buffer solution (pH 8.8) and finally stored in 25 mM Tris-HCL buffer (pH 8.8) at 4°C. Protein concentrations in the supernatant before and after immobilization were determined by the Bradford assay and the amount of enzyme immobilized within HM-PAL-CLEAs was calculated based on mass balance.

### Activity Assay

The activities of free PAL, PAL-CLEAs and HM-PAL-CLEAs were measured by a modified procedure [25], [27]. A small amount of enzyme sample was added to a reaction medium comprising of 25 mM Tris-HCl buffer solution (pH 8.8) supplemented with 50 mM L-phenylalanine. The resultant reaction medium was incubated at 30°C for 1 h. The reaction was terminated by addition of 6 M HCl. After centrifugation, the rate of formation of trans-cinnamic acids was determined by measuring the increase in A_278 nm_ with a 2800 H spectrophotometer (Unicoi Instrument Co., Ltd. Shanghai). One unit of PAL activity was defined as the amount of enzyme required to convert one µmole of L-phenylalanine to trans-cinnamic acids per minute. The activity assay of immobilized enzymes was detected by the same procedure as described above. The activity recovery in PAL-CLEAs and HM-PAL-CLEAs were calculated by comparing the specific activity of enzyme before and after immobilization. As given in Eq.1:
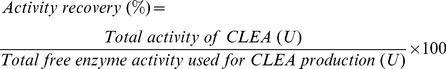
(1)


### Structural Characterization of HM-PAL-CLEAs

Morphology of the magnetite nanoparticles, PAL-CLEAs and HM-PAL-CLEAs was examined by scanning electron micrographs (SEMs) (JEOL JSM6700, Japan). The samples were dried at ambient temperature and then coated with platinum under vacuum. Confocal laser scanning microscopy (CLSM) was used to investigate the distribution of PAL within CLEAs. Prior to observation, the CLEAs samples were mixed with fluorescamine solution (50 mg/ml, Fluorescamine was soluble in acetone) for 3 min to form highly fluorescent product by the reaction between primary amines in proteins and the fluorescamine [29]. CLSM observation was performed with a Leica TCS SP5 microscope (Leica Camera AG, Germany). The samples were excited at 390 nm and the emitted fluorescent light was detected between 460 and 480 nm.

### Measurement of Kinetic Parameters

The kinetic parameters (*K_m_* and *V_max_*) for the free PAL, PAL-CLEAs and HM-PAL-CLEAs were determined by measuring the enzymatic reaction rates (using the same amount of enzyme) at different L-phenylalanine concentrations (from 5.0 mM to 150 mM). *K_m_* and *V_max_* were calculated from the Lineweaver-Burk equation using computed linear regression calculations.

### Effect of pH and Temperature on Enzyme Activity

The optimal pH for the free enzyme, PAL-CLEAs and HM-PAL-CLEAs was determined by adding the enzyme samples into the 50 mM L-phenylalanine solution with different pH values ranging from 6 to 11. The reaction was lasted for 30 min at 30°C. The optimal temperature of the free PAL, PAL-CLEAs and HM-PAL-CLEAs were determined by adding the enzyme samples into the 50 mM L-phenylalanine solution in 25 mM Tris-HCl buffer solution pH 8.8 at different temperatures (30–80°C) for 30 min. The activity of the free enzyme, PAL-CLEAs and HM-PAL-CLEAs was determined as mentioned in activity assay section.

### The Stability of Free Enzyme, PAL-CLEAs and HM-PAL-CLEAs

Thermal stabilities of the free enzyme, PAL-CLEAs and HM-PAL-CLEAs were determined by incubating them in 25 mM Tris-HCl buffer solution (pH 8.8) without substrate at 60°C for 20–100 min. At different incubation times, the residual PAL activity was determined by the same procedure as described above. The stability of enzymes against different denaturants including 3.0 M guanidine hydrochloride (GuHCl), or ethanol (20%, *v/v*) in 25 mM Tris-HCl buffer solution (pH 8.8) were also tested by measuring the residual activity of enzymes after being incubated in the respective denaturant solution for 1 h at 30°C. Storage stabilities of the free enzyme, PAL-CLEAs and HM-PAL-CLEAs were determined by incubating enzyme samples in 25 mM Tris-HCl buffer solution (pH 8.8) without substrate at 4°C or 25°C. At different storage times, free PAL, PAL-CLEAs and HM-PAL-CLEAs were separated and washed by distilled water. Then the residual PAL activities in these immobilized enzyme and free enzyme samples were determined.

### Reusability of CLEAs

Reusability of PAL-CLEAs and HM-PAL-CLEAs for biotransformation of trans-cinnamic acids to the L-phenylalanine was evaluated. The reaction system (1.0 ml) was comprised of 50 mg of CLEAs in 1 ml 10 g/L trans-cinnamic acid, and 25% ammonium hydroxide (pH 10.5). Each cycle was lasted for 1 h at 30°C, after that, the PAL-CLEAs were separated from the reactants by centrifugation and HM-PAL-CLEAs were separated by magnetic field, respectively. After thoroughly washing with buffer solution, the biocatalysts were re-suspended in a fresh reaction mixture for enzyme activity measurements as described in Activity Assay section. Thereafter, the biocatalysts were repeatedly used for the next reaction cycle. The residual PAL activity of each cycle was calculated by taking the enzyme activity of the first cycle as 100%.

### Statistical Analysis

All the experiments in this study were carried out in triplicates. The data were statistically analyzed according to SAS (1985). Significant differences between any two means were determined at the 5% level by using T-test.

## Results

The optimization of preparing HM-PAL-CLEAs was carried out by combination of traditional non-statistical technology and statistical technology based experimental design. First, the preparation of HM-PAL-CLEAs (one-factor-at-a-time experiments) was elucidated by non-statistical technology, the results revealed that the crude enzyme concentration, magnetite nanoparticles concentration and glutaraldehyde concentration were supposed to have effects on recovery activity (data not shown). Then, the Central Composite Design (CCD) was used to study the combined effect of the crude enzyme concentration, magnetite nanoparticles concentration and glutaraldehyde concentration for recovery activity according to central composite design (CCD) and response surface methodology (RSM), and to understand the relationship between the factors and recovery activity. The crude enzyme concentration (*X*_1_), magnetite nanoparticles concentration (*X*_2_) and glutaraldehyde concentration (*X*_3_) were chosen as the independent variables shown in Table 1. Recovery activity (*Y*) was used as dependent output variables. A set of 20 experiments consisting of 8 factorial points, 6 axial points and 6 replicates at the center points were employed. All experiments were carried out in triplicates. Amultiple regression analysis of the data was carried out with the statistical package (Stat-Ease Inc., Minneapolis, MN, USA). Table 2 showed the actual recovery activity in CCD. The regression coefficients and signficance levels were given in Table 3. Table 3 indicated that the quadratic term (*X*_2_, *X*_3_ and *X* ^2^_3_) were significant (“probe*>F*” less than 0.01), It means that magnetite nanoparticles concentration and glutaraldehyde concentration have important effects on PAL recovery activity, and the quadratic effect of glutaraldehyde concentration is more significant than other factors. but the interactions of three variables were not significant. It means that there were no interactions among three independent variables. Miltiple regression analysis of the experimental data gave the following second-order polynomial equation:
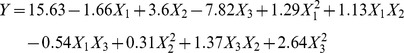
where *X*_1_, *X*_2_ and *X*_3_ are the crude enzyme concentration, magnetite nanoparticles concentration and glutaraldehyde concentration, respectively. The regression equation obtained from analysis of variance (ANOVA) indicated that the multiple correlation coefficient of *R* ^2^ is 0.9236. The value of the determination coefficent (*R* ^2^ = 0.9236) indicates that the model can explain 92.36% variation in the response, and only 7.64% of the total variations are not explained by the model. The value of adjusted *R* ^2^ (Adj. *R* ^2^ = 0.8549) is very high that indicated a high significance of the model. The model *F*-value of 13.44 implied that the model was significant. From the statistical results obtained, it was shown that the above models were adequate to predict recovery activity within the range of variables studied.

**Table 1:** Process variables used central composite design with actual factor levels corresponding to coded factor levels.

Variables	Symbol	Coded levels				
		−2	−1	0	1	2
Crude enzyme(mg/ml)	*X*_1_	0.9	1.8	2.4	3.6	4.5
Nanoparticles(mg/ml)	*X*_2_	8	18	24	32	40
Glutaraldehyde (v/v, %)	*X*_3_	0.05	0.1	0.15	0.2	0.25

**Table 2:** Central composite design and response value.

Run	*X*_1_	*X*_2_	*X*_3_	Recovery activity (%)
1	0	0	0	19.84
2	0	0	0	15.94
3	0	0	0	16.53
4	+1	+1	+1	14.99
5	+1	−1	+1	8.04
6	+1	+1	−1	31.43
7	0	−2	0	8.3
8	0	0	0	10.13
9	0	0	2	8.18
10	0	0	0	12.75
11	−1	−1	+1	9.89
12	0	0	0	17.13
13	−1	+1	+1	21.02
14	0	0	0	17.01
15	−1	−1	−1	29.62
16	0	0	−2	43.23
17	0	2	0	24.49
18	+1	−1	−1	21.26
19	−2	0	0	24.12
20	−1	+1	−1	26.60

**Table 3:** Parameter estimates and analysis of variance.

Source of variation	Degree of freedom (df)	Sum of squares (SS)	Mean squares (MS)	F-value	Pr>F
*X*_1_	1	44.19	44.19	3.69	0.0838
*X*_2_	1	207.43	207.43	17.30	0.0019**
*X*_3_	1	977.66	977.66	81.56	<0.0001**
*X*_1_^2^	1	42.16	42.16	3.52	0.0902
*X*_1_ *X*_2_	1	10.15	10.15	0.85	0.3792
*X*_1_ *X*_3_	1	2.37	2.37	0.2	0.6663
*X*_2_^2^	1	2.45	2.45	0.2	0.6606
*X*_2_ *X*_3_	1	14.93	14.93	1.25	0.2905
*X*_3_^2^	1	175.22	175.22	14.62	0.0034**
Model	9	1449.93	161.10	13.44	0.0002**
Residual	10	119.87	11.99		
Lack of fit	5	59.51	11.90	0.99	0.506
Pure error	5	60.36	12.07		
Correct total	19	1569.79			

**indicate highly significant.

The optimal conditions were extracted by Design Expert Software, the real values were the crude enzyme concentration at 2.1 mg/ml, magnetite nanoparticles concentration at 33.45 mg/ml, and glutaraldehyde concentration at 0.05% (v/v). The maximum recombinant PAL recovery activity obtained by using the above optimized concentrations of the variables is 43.27%. The maximum PAL recovery activity obtained experimentally was found to be 42% (PAL activity of HM-PAL-CLEAs is about 0.5 U/g). This is obviously in close agreement with the model prediction.

**Figure 1:**
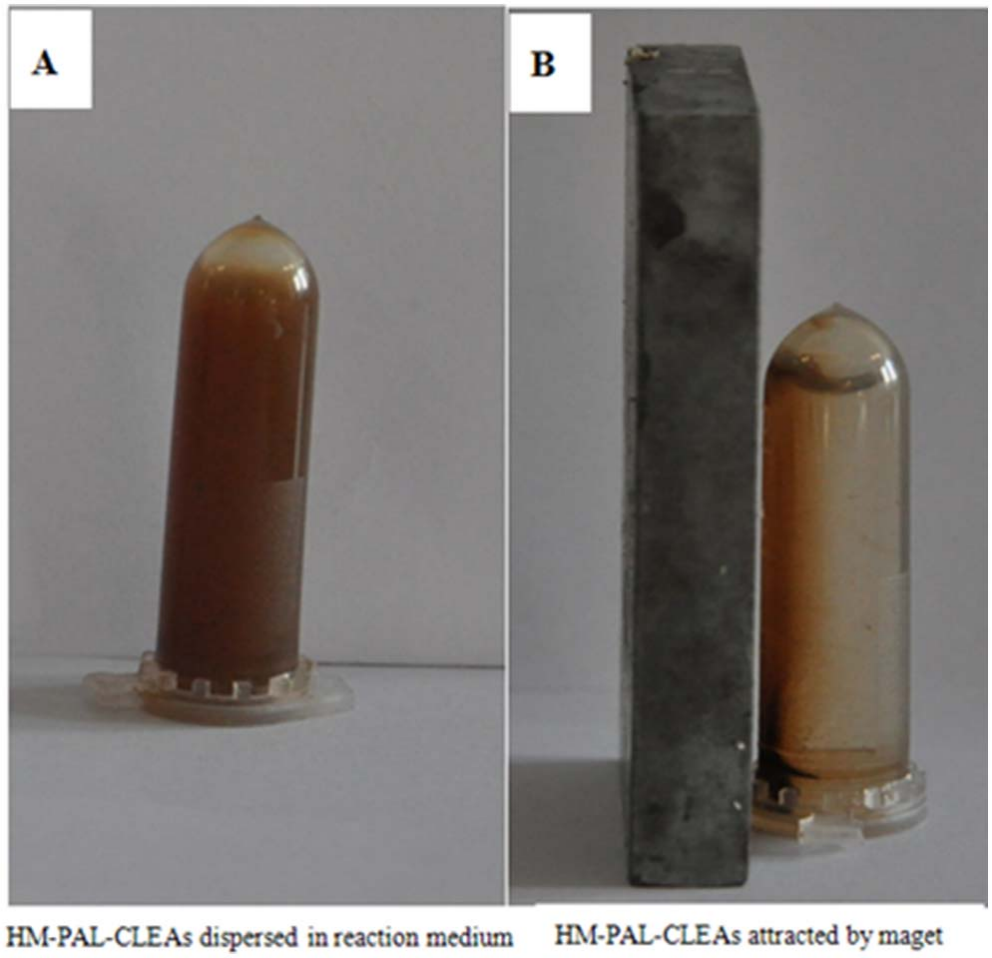
HM-PAL-CLEAs could be separated from reaction mixture by magnetic field. (A) HM-PAL-CLEAs were dispersed in reaction medium; (B) HM-PAL-CLEAs were concentrated and collected by an external magnet.

**Figure 2:**
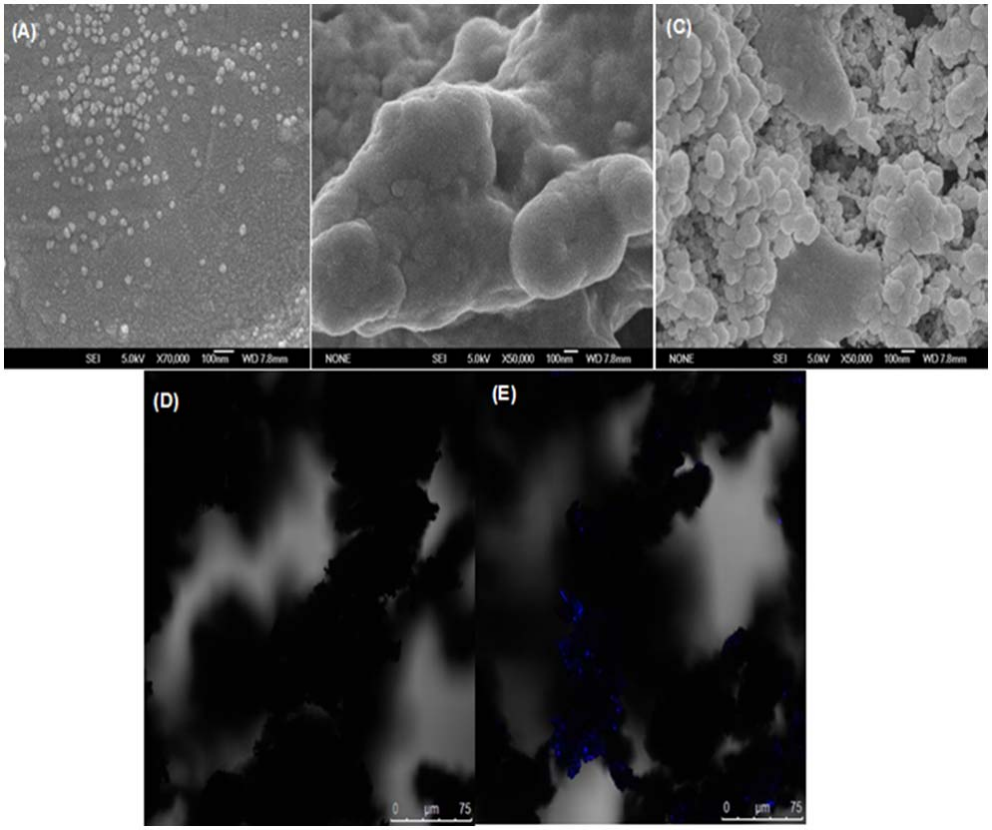
SEM images of (A) magnetite nanoparticles magnified 70,000×; (B) PAL-CLEAs magnified 50,000×; (C) HM-PAL-CLEAs magnified 50,000×; as well as LCSM images of (D) magnetite nanoparticles with fluorescamine treatment and (E) HM-PAL-CLEAs with fluorescamine treatment.

Magnetic response of the prepared HM-PAL-CLEAs was showed in Fig. 1. It can be seen that the HM-PAL-CLEAs could be easily separated from reaction mixture by exerting an external magnetic field. The SEM images of the as-prepared magnetite nanoparticles, PAL-CLEAs and HM-PAL-CLEAs are shown in Fig. 2. The magnetite nanoparticles remained discrete and were spherical in shape (Fig. 2(a)), while both PAL-CLEAs and HM-PAL-CLEAs showed irregularly morphology (Fig. 2(b) and 2(c)). Unlike traditional strategy in which enzymes are covalently immobilized on nanoparticles, in hybrid HM-PAL-CLEAs aggregates, enzyme aggregates formed by PAL cross-linking each other were inlayed within nanoparticles aggregates without forming any covalent bonds. To further confirm the embedding of PAL within the HM-PAL-CLEAs aggregates, a confocal laser scanning microscopy (LCSM) was employed to investigate the distribution. As shown in Fig. 2(d) and (e), nanoparticles aggregates without immobilized PAL had not fluorescent Fig. 2(d), whereas nanoparticles aggregates with embed PAL-CLEAs had a apparent fluorescent (Fig. 2(e)), indicating that the enzyme was embedded within the HM-PAL-CLEAs aggregates.

Kinetic parameters of free enzyme, PAL-CLEAs and HM-PAL-CLEAs were determined by measuring initial reaction rates for each form with varying concentrations of substrate. By Lineweaver-Burk plot of 1/*V* versus 1/[*S*], Michaelis–Menten constant of the enzymes were determined, and their corresponding values were listed in Table 4. *K_m_* values of all three forms are nearly equal, which indicated the enzyme affinity for the substrate was few changed after CLEAs and magnetic CLEAs preparation. In contrast, the higher *V_max_* value of HM-PAL-CLEAs than PAL-CLEAs indicated the advantageous of hybrid magnetic CLEAs strategy. The *V_max_* value of HM-PAL-CLEAs accounted about 60% of that of its free formations. The reduced *V_max_* value can be explained by the reduced flexibility and deformation of PAL enzyme molecules after being crosslinked, which resulted in difficult access of the substrate to the enzyme [15], [21].

**Table 4:** Kinetic parameters of free PAL, PAL-CLEAs and HM-PAL-CLEAs.

Form of PAL	*K_m_* (mM)	*V_max_* (µM/L.min)	R^2^
Free PAL	1.15±0.05	8.89±0.44	0.9967
PAL-CLEAs	1.24±0.06	2.19±0.11	0.9985
HM-PAL-CLEAS	1.16±0.05	5.01±0.25	0.9976

R is the correlation coefficient of the Lineweaver-Burke plot.

**Figure 3:**
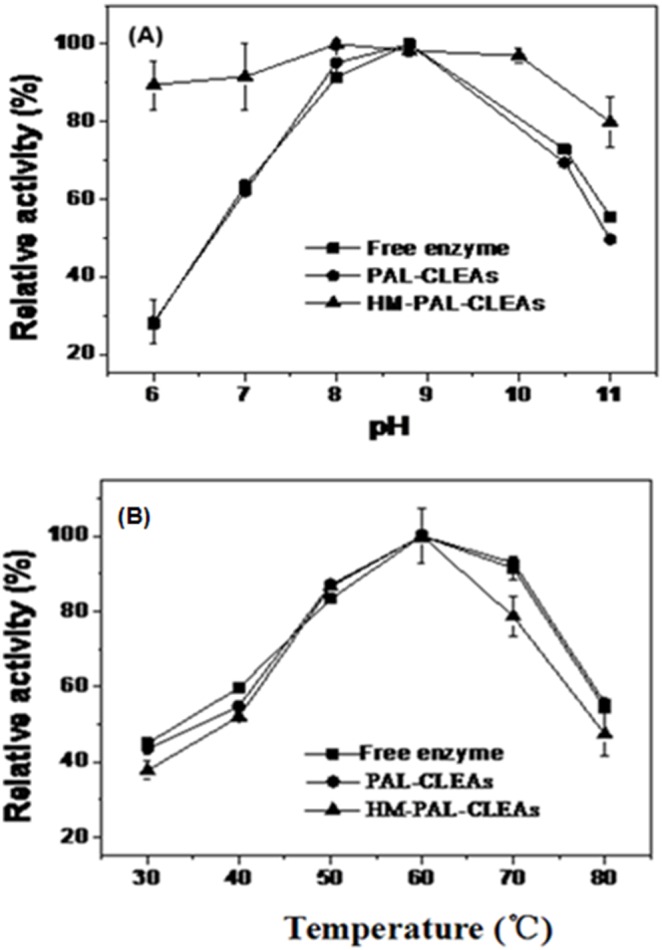
Optimal pH (A) and temperature (B) for free PAL, PAL-CLEAs, and HM-PAL-CLEAs. The activities are normalized relative to the highest activity value. Each bar represented the mean ± SD of three replicates.

The highest PAL activity in both free PAL and PAL-CLEAs was displayed at pH 8.8, whereas HM-PAL-CLEAs exhibited a much broader pH range of 8 to 10 with higher activity than free PAL and PAL-CLEAs (Fig. 3a). The broadened optimal pH range for high enzyme activity of the HM-PAL-CLEAs might be caused by the change in acidic and basic amino acid side chain ionization in the microenvironment around the active site [30], [31]. Fig. 3(b) showed the effect of temperature PAL enzyme activity. The results showed that within the tested temperature ranges, the dependence of activity of PAL, PAL-CLEAs and HM-PAL-CLEAs on temperature were similar, and all of them showed the highest activity at 55°C. The immobilized preparation did not increase the optimal temperature obviously. Similar results were observed in our previous reports when PAL was aggregated in microporous silica gel [32].

**Figure 4:**
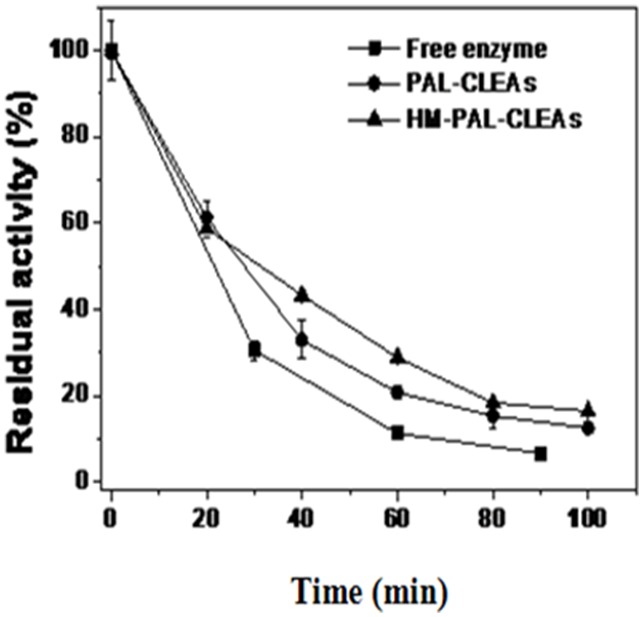
Thermal stability of free PAL, PAL-CLEAs and HM-PAL-CLEAs at 60°C. Each bar represented the mean ± SD of three replicates.

The thermal stability of free enzyme, PAL-CLEAs and HM-PAL-CLEAs at 60°C was shown in Fig. 4. Although the thermal stability of PAL for HM-PAL-CLEAs was not improved significantly compared to PAL-CLEAs (was only increased by 7%), HM-PAL-CLEAs showed the highest stability compared with the free PAL and PAL-CLEAs. Especially, HM-PAL-CLEAs retained more than 25% of their initial activity after 100 min incubation time at 60°C, whereas free enzyme lost most of activity in the same conditions. The results might be due to the insufficient covalent cross-linking between enzyme molecules and magnetite nanoparticles.

**Figure 5:**
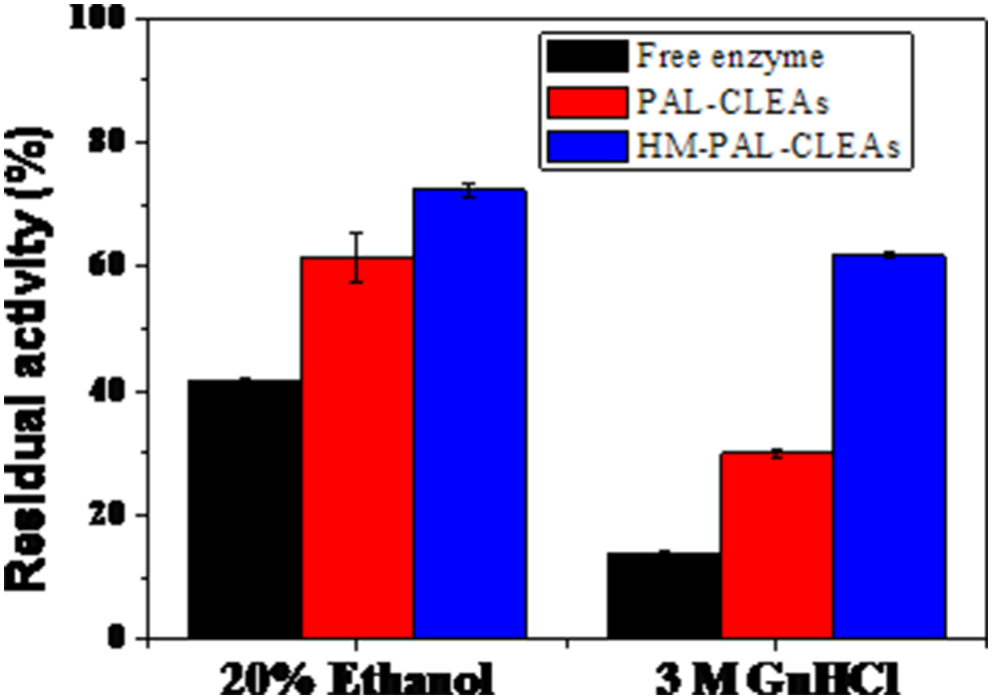
Stability of free PAL, PAL-CLEAs and HM-PAL-CLEAs against different denaturants. The activities are normalized relative to the initial activity. Each bar represented the mean ± SD of three replicates.

As is known, alcohol and guanidine hydrochloride are effective denaturants to proteins that can cause irreversible denaturation of protein. Fig. 5 showed the stability of free enzyme, PAL-CLEAs and HM-PAL-CLEAs in denaturants solutions. The results showed that both denaturants caused serous inactivation in enzymes in three formations, but PAL-CLEAs and HM-PAL-CLEAs obviously showed relatively better tolerant against this inactivation. In particular, after being incubated in the presence of 3 M guanidine hydrochloride, the free PAL showed almost no activity, while HM-PAL-CLEAs still retained 62% of its original activity, as compared to 30% of PAL-CLEAs.

**Figure 6:**
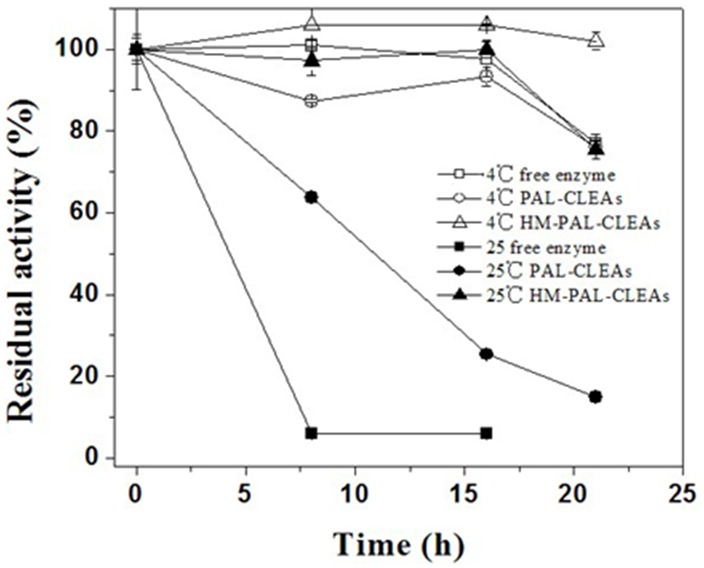
Storage stability of the free PAL (□, ▪), PALCLEAs (○, •) and HM-PAL-CLEAs (△, ▴) at 4°C (open symbols) and 25°C (filled symbols). The activities are normalized relative to the initial activity. Each bar represented the mean ± SD of three replicates.

The storage stability of the free PAL, PAL-CLEAs and HM-PAL-CLEAs was evaluated at 4°C and 25°C. Figure 6 showed that under both storage conditions, the results showed that free PAL are as stable as their CLEAs under 4°C tested before 16 days of storage. However, after 16 day, the activity of free PAL and PAL-CLEAs was reduced. Especially, the HM-PAL-CLEAs still retained almost 100% of its initial activity after being stored for 21 days at 4°C, while free PAL only retained about 77% of its initial activity. In contrast, at 25°C, the HM-PAL-CLEAs still retained high activity whereas the activity of free PAL and PAL-CLEAs decreased dramaticlly. Especially, the HM-PAL-CLEAs kept 75% of its initial activity at 25°C after 21 days of storage, whereas the free PAL lost all of its activity after the same storage period. Consequently, the present method of preparation of CLEAs conferred extended storage life to the enzyme.

**Figure 7:**
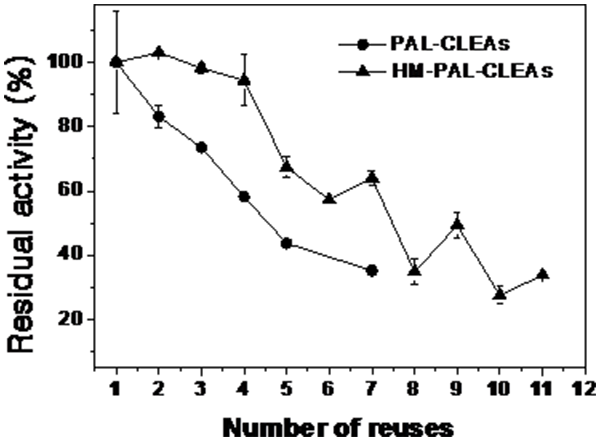
Stability of PAL-CLEAs and HM-PAL-CLEAs during reuse cycles. Each bar represented the mean ± SD of three replicates.

The duration of a catalyst is an important feature for its potential application in industry. The durability of PAL-CLEAs and HM-PAL-CLEAs for repeated batch biotransformation of trans-cinnamic acids to the L-phenylalanine was evaluated. As shown in Fig. 7, HM-PAL-CLEAs could be reused for 4 cycles without dramatic activity loss and still retained 30% of its initial activity after 11 cycles, whereas PAL-CLEAs retained 35% of its initial activity only after 7 cycles. The enhanced reusability of HM-PAL-CLEAs was speculated partially benefited from the milder operation of magnetic separating operation than centrifugation performed on PAL-CLEAs. The number of surface amine groups of PAL was not enough to achieve efficient cross-linking of all the enzyme molecules in CLEAs [18], [33], therefore leaking of enzyme from CLEAs would be a major reason for the loss in PAL activity. This problem will be more serous for CLEAs that need vigorous centrifugation for separation and shaking for re-suspension. On the other hand, the mild magnetic separation of HM-PAL-CLEAs would significantly reduce the dissociation of the aggregates, thus the leaking of enzyme from HM-PAL-CLEAs and the loss in enzyme activity during repeated usage would be reduced.

## Discussion

To date, carrier-free immobilization mainly includes cross-linked enzyme (CLEs), cross-linked enzyme crystals (CLECs), cross-linked spray-dried enzyme (CSDE) and cross-linked enzyme aggregates (CLEAs). CLEAs technology is attractive in its simplicity and robustness compared with other carrier-free immobilized enzymes technology [2], [4], [25], [31], however, some undesirable properties also limited its further application. Some reports showed that enzymes having low number of amino groups often undergo inadequate cross-linking and form mechanically fragile CLEA [3], [5], [8]. The other researchers found that the increased size of CLEAs clusters by centrifugation and filtration treatments causes internal mass-transfer limitations about special accessibility problem for macromolecular substrates [9], [11], [12], [32]. In order to solve these problems, some improving strategies of CLEAs technology were developed. Dong et al. prepared the CLEA of aminoacylase by using bovine serum albumin as an inert additive. The results showed that adding BSA dramatically improved the protein recovery, activity recovery and stability [3]. Wilson et al. found that the co-precipitation of the lipases with poly-ethyleneimine (PEI) or PEI-sulfate dextran (DS) mixtures permitted to get fully physically stable CLEAs [5]. However, the addition of these polymers and protein feeder results in the volumetric amount of immobilized enzyme decreases because these additives are not catalytically active. Jung et al. prepared CLEAs of glucose oxidase (GOx) in the pores of mesocellular foams (MCFs). The immobilized cross-linked GOx exhibited high activity and good mechanical properties. Furthermore, leaching was significantly reduced by the formation of GOx–CLEAs in the pores of the MCF support [13]. However, the volumetric activity of the biocatalyst can be decreased simply due to the presence of the carrier. Recently, the magnetic CLEAs were prepared by chemical cross-linking of enzyme aggregates with amino functionalized magnetite nanoparticles, the magnetic CLEAs exhibited good operational stability and magnetic response [21], [26]. It indicated that the strategy of magnetic CLEAs could make contributions in providing stable CLEAs preparation. In this report, we developed a hybrid magnetic CLEAs by co-aggregation of enzyme with magnetite nanoparticles and subsequent crosslinking with glutaraldehyde. The hybrid magnetic CLEAs differ from the traditional magnetic CLEAs. For preparation of traditional magnetic CLEAs, enzymes are usually immobilized on nanoparticles by ovalent bonds. Whereas, in hybrid magnetic CLEAs, enzyme aggregates formed cross-links one another and were inserted in nanoparticles aggregates without forming any covalent bonds (Fig. 2). Compared to non-magnetic CLEAs, the incorporation of magnetic nanoparticles not only facilitate the separation and recycling of the immobilized enzyme from reactant system (Fig. 1), but also enabled the immobilized PAL a broader optimal pH (Fig. 3), improved stabilities in storage (Fig. 6) and against denaturant agents (Fig. 5), and better reusability (Fig. 7). All these advantages of HM-PAL-CLEAs will open an attractive way towards stable CLEAs preparation. Compared to the preparation of traditional magnetic CLEAs, the strategy is simple and the cost of the method is low. This new technology may have potential for development as an efficient technology for preparing stable CLEAs. Taken together, these results mean that the hybird magnetic CLEAs technique could be used as a promising technique for improved CLEAs applications.
